# Testing the sequence of successional processes in miniature ecosystems

**DOI:** 10.1128/spectrum.01227-24

**Published:** 2024-08-27

**Authors:** Maximilian Hanusch, Xie He, Laura Böll, Robert R. Junker

**Affiliations:** 1 Evolutionary Ecology of Plants, Department of Biology, Philipps-University Marburg, Marburg, Germany; 2 Department of Environment and Biodiversity, Paris-Lodron-University Salzburg, Salzburg, Austria; Instituto de Ecología, A.C. (INECOL), Pátzcuaro, Michoacán, Mexico

**Keywords:** biotic interactions, dispersal, environmental filtering, artificial ecosystems, community assembly, succession, realism-precision

## Abstract

Dispersal, environmental filtering, and biotic interactions define the species inventory of local communities. Along successional gradients, these assembly processes are predicted to sequentially vary in their relative importance with dispersal as the dominating process early in succession, followed by environmental filtering and biotic interactions at later stages. While observational data from field studies supported this prediction, controlled experiments confirming a sequence of successional processes are still lacking. We designed miniature ecosystems to explicitly test these assumptions under controlled laboratory conditions. Our “Ecosystems on a Plate” (EsoaP) are 3D-printed customized microplates with 24 connected wells allowing us to track dispersal, niche filtering, and biotic interactions among bacteria and plants in time and space. Within EsoaPs, we created heterogeneous habitat landscapes by well-specific nutrient levels or by providing plant seedlings as mutualistic partners in a checkerboard pattern. Bacteria of a single strain were released in one well and subsequently distributed themselves within the plates. We measured the spatial distribution of bacterial abundances at two time points as a function of abiotic or biotic heterogeneity. Bacterial abundance distribution confirmed a shift from initial dispersal-dominated processes to later niche filtering and biotic interactions as more important processes. Our approach follows the principles of *open science* as the affordable availability of 3D printers as well as shared STL files makes EsoaPs disseminatable and accessible to all levels of society, facilitating future experimental research.

**IMPORTANCE:**

Hypotheses regarding the underlying processes of ecological successions have primarily emerged from and have been tested in observational studies, lacking substantial support through controlled experiments. The design of such experiments should focus on testing contemporary ecological theories at the intersection of community assembly and successional research. To achieve this, we developed and employed 3D-printed “Ecosystems on a Plate” (EsoaP) within controlled laboratory settings. EsoaPs surmount several limitations of nanoscale instruments that had hindered their application in ecologically meaningful research. By sharing 3D printing designs, experimental protocols, and data openly, we facilitate reproducibility of our experiments by researchers across diverse ecological disciplines. Moreover, our approach facilitates cost-effective replication of experiments, democratizing access to tools for ecological research, and thus holds the potential to serve as a model for future studies and educational purposes.

## INTRODUCTION

Ecological successions are community assemblies in progress. However, the underlying processes of community assembly (i.e., dispersal, niche filtering, and biotic interactions) have only rarely been described and tested in the context of successional theory (1–4). Recent conceptual frameworks predict these assembly processes to sequentially vary in their relative importance with dispersal as the dominating process early in succession, followed by environmental filtering and biotic interactions at later stages (1). Studies based on observational field data support these frameworks and identified sequential shifts in the main drivers of local community assembly from a primarily dispersal-dominated assembly to environmental filtering and biotic interactions as main assembly processes later in succession (5–9). Yet, a validation of these assumptions using controlled experiments is still lacking (10, 11). Insights originating from ecological research, whether through field observations or experiments, manifest along a continuum bridging realism and precision. Although field observations maintain ecological authenticity and exhibit a high degree of realism, the absence of controlled conditions can introduce challenges when attempting to validate hypotheses or even result in erroneous assumptions regarding ecological patterns as they tend to reveal correlations rather than establish causal relationships (12, 13). Further, it has been convincingly demonstrated that subjective decisions in how to analyze observational field data lead to substantial variation in reported effect sizes and even to contrasting qualitative conclusions (14). Thus, next to sampling errors and noise in field data, statistical decisions may prevent the inference of causality or a clear mechanistic understanding of ecological processes. This inaccuracy inherent to field observations emphasizes the importance of experimental confirmations to support and substantiate ecological conclusions. Laboratory experiments, on the other hand, prioritize controlled settings to pinpoint specific causal mechanisms, albeit at the expense of the authenticity found in field observations. Yet, controlled experiments allow us to add causality to the correlative findings from the former approach and foster mechanistic insights into successional processes (15). They provide a level of precision that is not achievable through observational studies alone and allow us to investigate ecological processes within spatial and temporal scales that are much smaller and shorter than their natural ecosystem counterparts, enabling researchers to validate and refine the findings obtained from field observations and statistical analyses.

**Fig 1:**
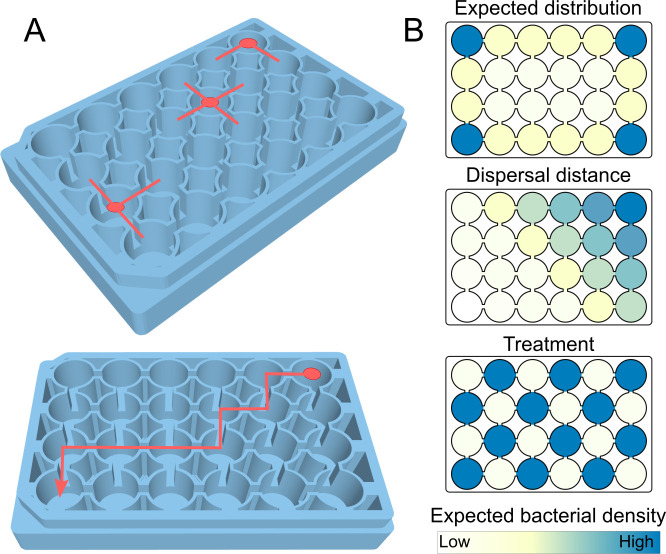
Ecosystems on a Plate (EsoaPs). (**A**) Technical drawing of EsoaPs. The upper drawing depicts the variation in the number of connected wells in corners, edges, and the center of EsoaPs. The lower drawing depicts Manhattan distances from the release well. (B) Schematic representation of the three null-model expectations. Expected bacterial densities are color coded from low densities (white to yellow) to high densities (blue). Upper graph: based on empirical observations of pre-trials, we expect bacterial densities to be higher in wells with less connections to adjacent cells and at the edges of the plate. Center graph: with an increasing distance from the initial well, bacterial densities are expected to decrease. Bottom drawing: nutrients and plants as mutualistic partners are arranged in a checkerboard distribution. We expect bacterial densities to be higher on treated wells.

In this study, we designed miniature ecosystems “Ecosystems on a Plate” (EsoaP; Fig. 1A) to explicitly test the sequential shift of assembly processes under controlled laboratory conditions. EsoaPs are 3D-printed customized microplates with 24 connected wells allowing us to track dispersal, niche filtering, and biotic interactions among bacteria and plants in time and space. EsoaPs overcome various aspects of nanoscale instruments that hampered their use for ecological meaningful research such as the inability to create chemical gradients, the limitations to culture only a small number of bacterial cells, and the missing possibility to grow plants within the experimental setup (16). Especially, if the aim is to support field-derived results with experimental evidence, laboratory experiments must be designed with the right balance between the simplicity needed to sustain experimental control while preserving an adequate representation of the complexity observed in the field. Within the EsoaPs designed for this study, bacteria are dispersed freely among fully connected wells using a platform shaker. We applied two treatments in a defined spatial order allowing us to examine dispersal, as well as the two further assembly processes via the spatial patterns of colonization (Fig. 1B): the first treatment tested for the process of environmental filtering by adjusting the nutrient content of specific wells. The second treatment focused on biotic interactions by the availability of plant seedlings that function as mutualistic partners providing necessary carbon to the bacteria as root exudates. In parallel, we performed experiments with homogenous distribution of nutrients as control. This setup enabled us to investigate the impacts of environmental filtering and biotic interactions on bacterial abundances of a field-collected bacterial strain from the genus *Rhodococcus* at two distinct time points (12 h and 36 h after bacterial release), capturing the dynamics of succession while considering potential dispersal limitations.

We established three null-model expectations for the distribution of bacterial density based on potential successional processes and compared the spatiotemporal patterns of bacterial growth to the control plates. The null-model expectations were defined a priori and translated into numerical values to serve as explanatory variables in our analysis using linear-mixed models as follows:

1. Expected density distribution (empirical observation)—in our system, bacterial dispersal is facilitated by rotating the EsoaPs. Accordingly, pre-trials showed that bacterial density was highest at the corners, medium in the edges, and lowest in the center of the EsoaP. This pattern corresponds to the number of connections to adjacent wells (i.e., wells in the corners have two connections, wells at the edges have three connections, and wells in the center of the plate have four; Fig. 1B, top). We thus expected bacterial densities to be negatively proportional to the number of connections of the wells.
2. Dispersal (distance)—bacteria were released in the top right well on each plate. Alternatively to the expectation explained above, bacteria may gradually disperse from well to well resulting in a decreasing density of bacteria with increasing Manhattan distance to the release well (Fig. 1B, center).
3. Niche effect—we arranged plant seedlings and nutrients in a checkerboard distribution within the plates to examine niche effects on bacterial densities. We expected that bacterial densities would be highest in wells with added nutrients or plants, resulting in a spatial pattern of bacterial density that reflects benign environmental conditions or the presence of a mutualistic plant partner (Fig. 1B, bottom).

## RESULTS

We empirically tested the hypothesized sequential changes of main assembly processes choosing a bacterial strain from the genus *Rhodococcus as* well as seeds of the plant species *Trifolium badium* as model organisms representing taxa involved in successional processes in a glacier forefield (17). The spatiotemporal distribution of the bacterial strain was tracked in a controlled laboratory setup using 3D-printed microcosm *ecosystems on a plate* (EsoaPs; Fig. 1A). The EsoaPs match the dimension of standard 24-well plates, compatible with most laboratory devices and can be easily replicated and adjusted using the digital blueprint (Supplemental Information 1). Within EsoaPs, we created heterogeneous habitat landscapes by well-specific nutrient levels or by providing plant seedlings as mutualistic partners in a checkerboard pattern. Bacteria were released in one well and subsequently distributed themselves within the plates. We measured their spatial distribution at two time points as a function of abiotic or biotic heterogeneity. Bacterial abundance distribution confirmed a shift from initial dispersal-dominated processes to later niche filtering and biotic interactions as more important processes.

**Fig 2:**
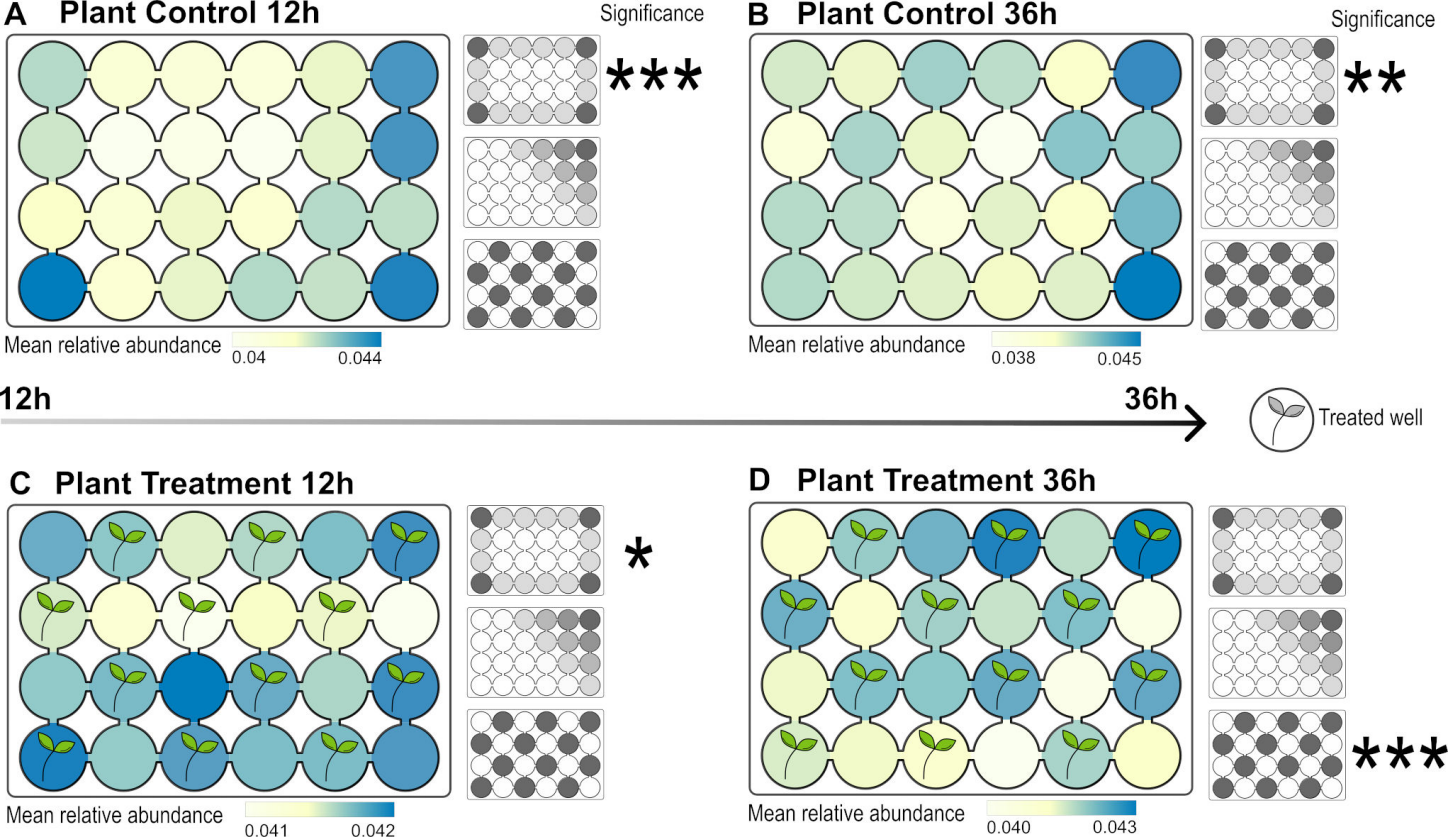
Mean relative bacterial densities in control (**A, B**) and treatment (**C, D**). Ecosystems on a Plate (EsoaPs) after 12 h (**A, C**) and 36 h (**B, D**) using nutrients to simulate environmental heterogeneity (**C, D**). Measured bacterial densities are color coded from low densities (yellow) to high densities (blue). The smaller plate drawings in grayscale represent the null models (i.e., expected density distribution, top; dispersal distance, center; and treatment, bottom). Significant effects of the null-model expectations are marked with asterisk. Patterns of bacterial densities on plates are best explained by (A) control 12 h, expected density distribution; (B) control 36 h, expected density distribution; (C) treatment 12 h, expected density distribution and treatment; and (D) treatment 36 h, expected density distribution, dispersal distance, and treatment.

**Fig 3:**
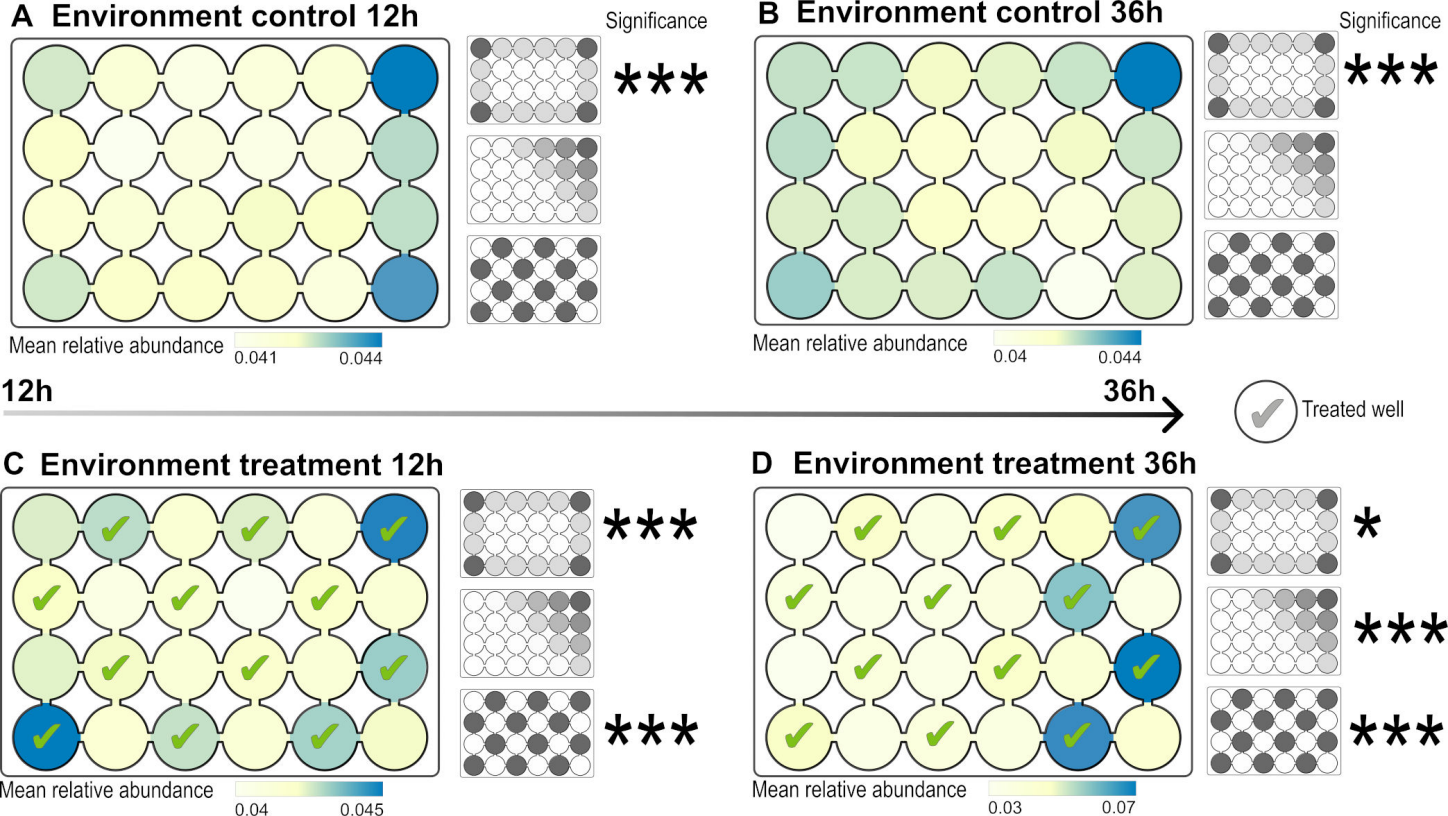
Mean relative bacterial densities in control (**A, B**) and treatment (**C, D**). Ecosystems on a Plate (EsoaPs) after 12 h (**A, C**) and 36 h (**B, D**) using a mutualistic partner to simulate spatially heterogeneity in the availability of mutualists (**C, D**). Measured bacterial densities are color coded from low densities (yellow) to high densities (blue). The smaller plate drawings in grayscale represent the null models (i.e., expected density distribution, top; dispersal distance, center; and treatment, bottom). Significant effects of the null-model expectations are marked with asterisk. Patterns of bacterial densities on plates are best explained by (A) control 12 h, expected density distribution; (B) control 36 h, expected density distribution; (C) treatment 12 h, expected density distribution; and (D) treatment 36 h, treatment.

Twelve hours after bacterial release, the distribution of bacterial density corresponded to the expected density distribution with highest densities in the corners, medium at the edges, and low in the center of the EsoaPs (expectation 1; Fig. 2A and C; Fig. 3A and C), indicating dispersal as the main process in the initial phase of the experiment. This effect was evident in control EsoaPs as well as in those treated with nutrients or plants. After 36 h, dispersal as single process shaping bacterial density was still evident in the control EsoaPs, i.e., in the absence of environmental filtering or biotic interactions (Fig. 2B and 3B). In contrast, bacterial density approached a checkerboard distribution after 36 h in EsoaPs with added nutrients (Fig. 2D) or plants (Fig. 3D), matching the third expectation indicating niche filtering and/or biotic interactions as dominant processes in later phases of the experiment (also see Supplemental Information 2).

## DISCUSSION

Community assembly processes are notoriously hard to identify from field-collected occurrence or abundance data (18). Thus, complementing field observations with controlled experiments is required to assess the relative importance of assembly processes along successions and to confirm contemporary ecological theories at the interplay of community assembly and successional research (10, 13, 15). Bacterial cultures in microcosms have been proven to be an excellent tool for ecologists to study community assembly or ecological successions and to evaluate theoretical predictions of ecological dynamics while avoiding noise typically found in field-based observational chronosequence approaches (19–22).

In our experiments, we simulated successional processes of bacterial colonization and related it to environmental heterogeneity, the presence of mutualistic plant partners, and spatial distance from the source population, i.e., dispersal. Our results confirmed observational findings from a field study (5) identifying dispersal, i.e., the ability of species to reach and colonize spaces, as the main process shaping the distribution of organisms early in succession. In later successional stages, abiotic factors and biotic interactions with other organisms become more important in defining the distribution of bacterial densities both in the field and in our experimental setting. These deterministic processes exert selective pressures on microbial communities and thus markedly affect the successional trajectories of taxa (23–25).

In the environmental treatment, we found significant effects of environmental filtering already after 12 h, whereas for the plant treatment, we did not find effects of biotic interactions at the early time point. Many bacteria follow opportunistic growth dynamics along successions with bacterial growth being primarily fueled by substrate-derived carbon sources in early successional stages (26). Later in succession, the main carbon source can be expanded or substituted by organic carbon supplied by external inputs, such as plant exudates (27). Thus, the detected temporal differences in growth patterns of bacteria may be attributable to a delayed availability of plant-based carbon in the plant treatment whereas the nutrient-medium-derived carbon is instantly available for consumption in the environmental treatment.

After 36 h, significant effects of all three null-model expectations were detectable in the environmental treatments, indicating legacy effects of dispersal distance. Here, the impact of the past dispersal event persists through time in the spatial distribution of bacterial densities, although the effects of environmental filtering on the distributional pattern of bacterial densities at a later stage of the experiment become more prominent. The plant treatment, in contrast, completely overrode the dispersal legacy after 36 h, indicating that trophic partners may favor establishment more strongly than the availability of environmental nutrients. Legacy effects have been shown to put important constraints on microbial successions by influencing the composition and function of microbial communities over time (28–30). Our findings highlight the need for careful consideration of past dispersal events when studying microbial community dynamics and legacy effects in natural settings.

Our experiments allowed us to trace a sequential change in the importance of assembly processes that is in line with the hypothesized framework of succession of Chang and HilleRisLambers (1) and correlative findings from field-based studies (5, 31). Although most theoretical work on the varying importance of dispersal and biotic interactions were centered around plants (27, 32), the importance of dispersal limitation has been claimed to be a common pattern that applies to various organismal groups other than plants such as microbes (5, 6). Given the important ecological and biogeochemical roles of microbes in natural ecosystems, our results advocate for further experimental investigation into the mechanisms underlying succession and community dynamics of microbes and their complex coactions with the environment and other organismal groups. Such interdisciplinary approaches will provide a more comprehensive understanding of ecosystem functioning and help to bridge the gap between microbial and ecosystem ecology.

Our study is a first test of the EsoaPs and proves their usability as a powerful tool for experimental ecological research. In this study, we only used a single bacterial strain tested either in an heterogenous environment or in combination with plant seedlings as second organisms. Although founded by the same clone and growing under identical environments, replicate populations from single bacterial strains often exhibit, albeit within limited ranges, divergence in various traits, including growth rates or interaction capabilities with other organisms or the environment (33). Naturally formed microbial communities rarely contain only one strain of bacteria (34); thus, our approach clearly is a simplification of the complexity inherent to natural ecosystems. However, in experimental approaches that compare the performance of single microbial strains in isolation versus within communities of multiple strains, the strain under study often exhibits varying responses when grown in multi-strain mixtures, complicating the identification of the acting processes on community assembly (35). Using only a single bacterial strain, our setup ensures the precision necessary for reliable laboratory measurements to assess the importance of individual assembly processes. While we did not specifically test for intraspecific variation in our study, future research could benefit from measuring this variability in response to different processes using environmentally standardized observational assays and protocols, such as EsoaPs. This would provide a promising avenue to further understand the complexities of microbial interactions and community assembly.

Our current configuration of the EsoaPs not only focuses on the effects of environmental filtering and biotic interactions on bacterial densities as a proxy for community assembly over time but also allows us to track dispersal and its impact on the trajectory of community assembly. Similar to most other ecological experimental setups, EsoaPs investigate ecological processes within spatial and temporal scales that are much smaller and shorter than their natural ecosystem counterparts (36). However, well-designed small-scale experimental systems allow us to give the processes of interest a realistic representation and are thus a valid tool for answering large-scale ecological questions (37). Well-designed experimental systems are thus a valuable tool for understanding ecosystem formation, as they provide a realistic representation of key processes, enabling researchers to address ecological questions about community development and dynamics (36, 37). EsoaPs incorporate the benefits of microbiological *in vitro* experiments while maintaining precise spatiotemporal control over the microenvironments (38–43). Researchers can build upon the current design or easily adapt the digital blueprint of EsoaPs to address manifold research questions that scope beyond bacterial community assembly. Customized modifications will allow to suit the specific needs of their experimental endeavors and could, for instance, expand the experimental design of EsoaPs to include the analysis of bacterial interactions or associated effects on plant-environment-microbe interdependencies, as well as testing bacterial meta-community and meta-population concepts. This flexibility is an example of how *open science* (44) can be promoted and allows for continued refinement and improvement of the EsoaPs. Thus, EsoaPs offer a straight-forward way of recreating biological environments in addition to other traditional micro- or nanoscopic cultures on fluidic wafers, droplets, or lab-on-a-chip culture systems (38, 40, 45) and may serve as a potential model system for future studies and teaching.

## MATERIALS AND METHODS

### Ecosystem on a plate

The plates' dimension matches the measures of standard 24-well plates and thus fits in most technical devices already present in many laboratories. EsoaPs can be easily (re-)produced using a conventional 3D printer following a standardized blueprint and easily adapted to fit the specific needs of various experimental setups (see Supplemental Information 1 for the technical drawing and construction plan as a 3D-printable STL file). Within the EsoaPs, bacteria are freely dispersed (using a platform shaker) while plants act as mutualistic partners for biotic interactions and modified nutritional levels in the wells reflect environmental variation. EsoaPs allow us to accurately simulate complex natural environments, mimic variations in environmental conditions, provide the opportunities to include biotic interactions, and analyze spatially explicit dispersal processes as opposed to other setups. For our experiments, we printed a total of *n =* 84 plates using a Mega X Printer (Anycubic, Shenzhen, People's Republic of China) following the construction plan that can be found in the supplementary information. For our experiments, a polylactic acid filament printed on a 0.4 mm nozzle with a layer height of 0.2 mm at an extruder temperature of 250°C and a printing speed of 60 mm/s proved to be the most suitable.

### Biotic sampling

In 2019, we established an alpine research platform along the chronosequence of the Ödenwinkel glacier in the Hohe Tauern National park (5, 17, 46). For our experiments, we field-collected seeds of the plant species *Trifolium badium* and isolated a bacterial strain belonging to the genus *Rhodococcus* from its phyllosphere microbiome. While *T. badium* is a widespread species along the successional gradient of the Ödenwinkel forefield (17), the genus *Rhodococcus* is a widespread bacterial taxon whose strains occur in soil as well as in associations with plants (47). It has been shown that many isolated bacterial strains are opportunistically able to grow using either abiotic resource from the environment or biotic resources from a synergistic community with plant partners (48).

### Isolation of bacterial strains

We field-collected, isolated, cultivated, and identified bacteria associated with the phyllosphere of *T. badium* by transferring plant tissues into a 50:50 buffer to glycerol solution and storing these samples at −80°C until further use. In order to increase survival possibilities for the collected microorganisms, samples taken were duplicated and kept both in LB medium and PBS buffer. Bacterial strains were isolated, and a collection of living organisms was created as described in reference (49). Isolated bacterial strains were identified by Sanger sequencing using the 16S rRNA marker (Aler1_341f: CCTACGGGAGGCAGCAG). For the taxonomic classification of the isolated bacteria, all sequences were manually trimmed using FinchTV 1.4.0 (Geospiza, Inc., Seattle, WA, USA). Afterwards, we used RDP's classifier (50) to perform a taxonomic placement of the sequences with a confidence threshold of 80% (for classification to Root ONLY) (51). Cultures, along with taxonomic classifications and sequences, were deposited at Herbarium Marburgense.

### Selection of bacterial strains

For our experiments, we screened the isolated bacterial strains for their performance under modified nutritional conditions and under the presence of a mutualistic plant partner. The aim was to identify a strain that showed only little or any growth under reduced nitrogen levels in the culture medium but responded positively to the presence of plant root exudates under the exclusion of other carbon sources. To identify potential strains, we tested *n* = 13 bacterial strains in trial runs for their performance in standard M9 culture medium without any ammonium chloride NH_4_Cl additions. Bacterial performance was tested using 96-well plates and calculating strain-specific growth curves in a plate reader for 7 days (Supplementary Information 3 and 4). Additionally, we germinated surface-sterilized seeds of *T. badium* and placed them onto petri dishes that were filled with M9 culture medium without glucose. We added bacteria into the vicinity of the seedlings to assess whether they can metabolize the root exudates as the carbon source. We then visually checked the plates for the initial formation of bacterial colonies along the roots of the plant. The bacterial strain that proved suitable for our experiments was identified to belong to the genus *Rhodococcus* using RDP's classifier with an 80% threshold (Supplementary Information 5).

### Experiments

First, EsoaP plates were thoroughly washed with a brush and detergent under hot water to remove any adherent coarse particles and other remains that could cause contaminations. Second, the plates were sterilized by submerging in 70% ethanol in a sterile 5 L container. Plates were kept in ethanol for 2 min and were gently shaken every 30 s to avoid trapped air bubbles. Subsequently, the plates were placed on a sterile bench to allow the remaining ethanol to evaporate. After drying, each well on the plates was filled with 1 mL agar of desired nutrient strength for the specific treatment and set aside covered with a sterile lid until the agar solidified. Third, bacterial colonies were picked from a growing plate (full strength M9 medium) and nutrient remains were removed by washing bacterial colonies in 150 µL nutrient-free PBS solution followed by centrifuging the bacterial solution. The washing step was repeated five times re-using the concentrated bacterial pellets that formed after centrifugation. The washed bacterial solution was adjusted to an optical density of 0.01 and 10 µL of the adjusted solution was transferred into the top right well of each plate. A total of 1 mL PBS solution was pipetted into the bottom left corner of each plate to serve as a dispersal medium on top of the solidified agar. All plates were then carefully placed on a shaker running at 120 rpm to initiate the passive dispersal of bacteria within the EsoaPs.

### Treatments

For the environmental filtering treatment, we used a full strength M9 growth medium without any NH_4_Cl additions to the medium and added NH_4_Cl only to selected wells within EsoaPs in a checkerboard distribution. For the biotic interaction treatment, we used sterile seedlings of *T. badium* on a full strength M9 medium without any glucose additions. In our treatments, the omission of NH_4_Cl resembles environmental variation (environmental filtering through nutrient availability) while plants act as mutualistic partners under a reduced carbon environment (plants provide necessary carbon as root exudates). Sterile seedlings of *T. badium* were grown from surface-sterilized seeds. The seed sterilization was performed by covering seeds in a 1:10 mixture of 13% sodium hypochlorite solution to autoclaved water. Seeds were vortexed for 1 min followed by an incubation of 15 min within the bleach solution. Seeds were then washed three times with sterile water while vortexing for 1 min and placed for germination on plain, sterile agar plates in a climate chamber at 16°C with artificial lighting under a 16/8 day-night cycle. Seven days after germination, individual seedlings were picked and transferred to the EsoaPs on selected wells in a checkerboard distribution as a treatment. To correct for potential mechanical retention and accumulation of bacteria along the stems of the plants during the dispersal phase within the EsoaPs, we placed sterilized pieces of string (ca. 1.5 cm in length, 1 mm diameter) in a checkerboard distribution to the EsoaPs of the control group to mimick the stem of the plants.

### Analysis of bacterial density

To assess bacterial density on each plate, we scraped along the inner edges of each well with a flat spatula to loosen the agar from the edges of the wells. The small agar droplets were then carefully lifted from below to avoid touching the bacterial cultures growing on top of the agar and transferred into fresh, sterile, conventional 24-well plates without connections (Greiner Bio-One, Austria) in the same order as they were arranged within the EsoaP. A total of 500 µL PBS solution was pipetted onto each agar droplet and the plates were placed on a shaker for 30 min at 500 rpm to wash the bacteria off the agar. The agar pieces were then carefully removed from the PBS solution that contained the bacteria with sterile tweezers and care was taken that all the PBS solution dripped off the agar pieces. A total of 200 µL of this bacterial solution was then transferred onto a fresh 96-well plate to estimate bacterial densities (optical density of 600) on a plate reader.

### Statistical analysis

Our experiments investigate the distribution of bacteria at two different time points (i.e., after 12 h and 36 h of bacterial growth) and thus allow us to test for the predicted temporal sequence of assembly processes. To allow for comparisons among treatment and control groups, as well as among plates, we used relative instead of absolute bacterial densities. Relative density was calculated for each EsoaP individually by dividing the measured density of a single well by the sum of all bacterial densities of all wells within the plate. To assess effect sizes of the null hypotheses, we included them in our analysis by using Manhattan distances for dispersal distance, zero and one coding for treatment application (i.e., whether a specific well contained NH_4_Cl or a plant seedling), and number of wells (as a surrogate for spatial position of the wells on the plate) as expected bacterial densities. In pre-experimental runs, the pattern of spatial position of a well showed a high correspondence to the number of connections to adjacent wells (i.e., wells in the corners have two connections, wells at the edges have three connections, and wells in the center of the plate have four; Fig. 1B). We thus expected bacterial densities to be negatively proportional to the number of connections of the wells.

We then used the relative densities to run linear-mixed models (R-package lme4 v1.1-28) (52) for each treatment and control run (i.e., *n =* 7 plates each for plant treatment and control and *n* = 14 plates each for environmental treatment and control after 12 h and 36 h). The three null hypotheses were integrated into the model as fixed effects, individual plates as random factor, and relative bacterial density as dependent variable. We assessed significance via a Type II analysis of variance (R-package car v3.0-1.2) (53) of the regression output.

## Data Availability

A 3D-printable model of the Ecosystems on a Plate used in this study can be found in the supplementary files. This paper does not report original code.

## SUPPLEMENTAL MATERIAL

Supplemental material spectrum.01227-24-s0001.pdf Supporting results and link to digital blueprint for EsaoP.

Supplemental data spectrum.01227-24-s0002.txt SI5_DNA_Sequence.fasta.

ASM does not own the copyrights to Supplemental Material that may be linked to, or accessed through, an article. The authors have granted ASM a non-exclusive, world-wide license to publish the Supplemental Material files. Please contact the corresponding author directly for reuse.
