# Supplementary material for: Testing the sequence of successional processes in miniature ecosystems
Source: Microbiol Spectr. 2024 Aug 27;12(10):e01227-24. doi: 10.1128/spectrum.01227-24 (PMC11448199; doi:10.1128/spectrum.01227-24)
Supplement: Supplemental material — Supporting results and link to digital blueprint for EsaoP. [file spectrum.01227-24-s0001.pdf]

Supplemental Information

Supplemental Information 1: Digital, 3D-Printable model of the Ecosystems on a Plate used in this study. File can be found under:

DOI:10.17632/fsbkbm8jwz.1

Supplemental Information 2: Output of the linear mixed models performed in this study. Table can be found in a separate document.

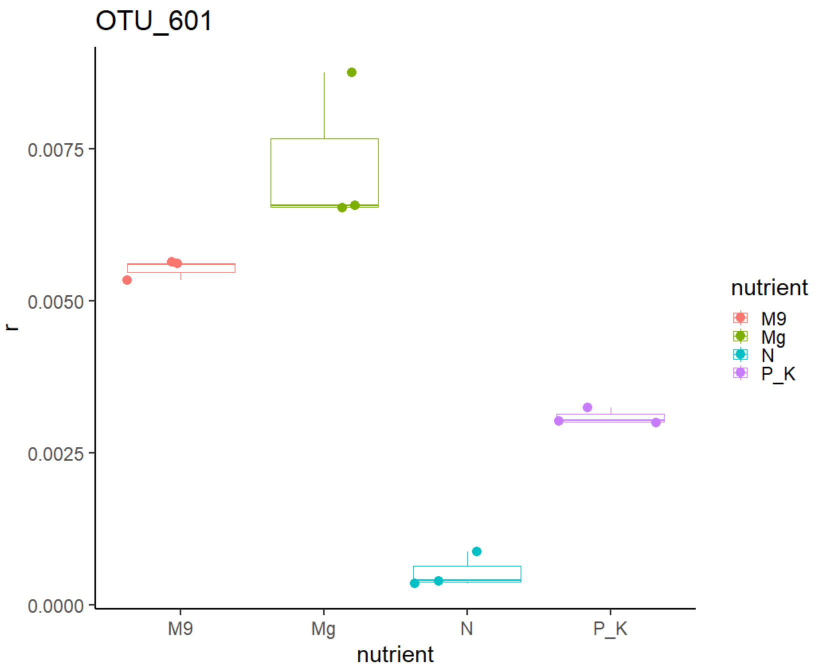

Supplemental Information 3: Growth rate of the selected strain of the genus *Rhodococcus* under nutrient scenarios during pre-experiments. Growth rate r is lowest under non-N-scenario.

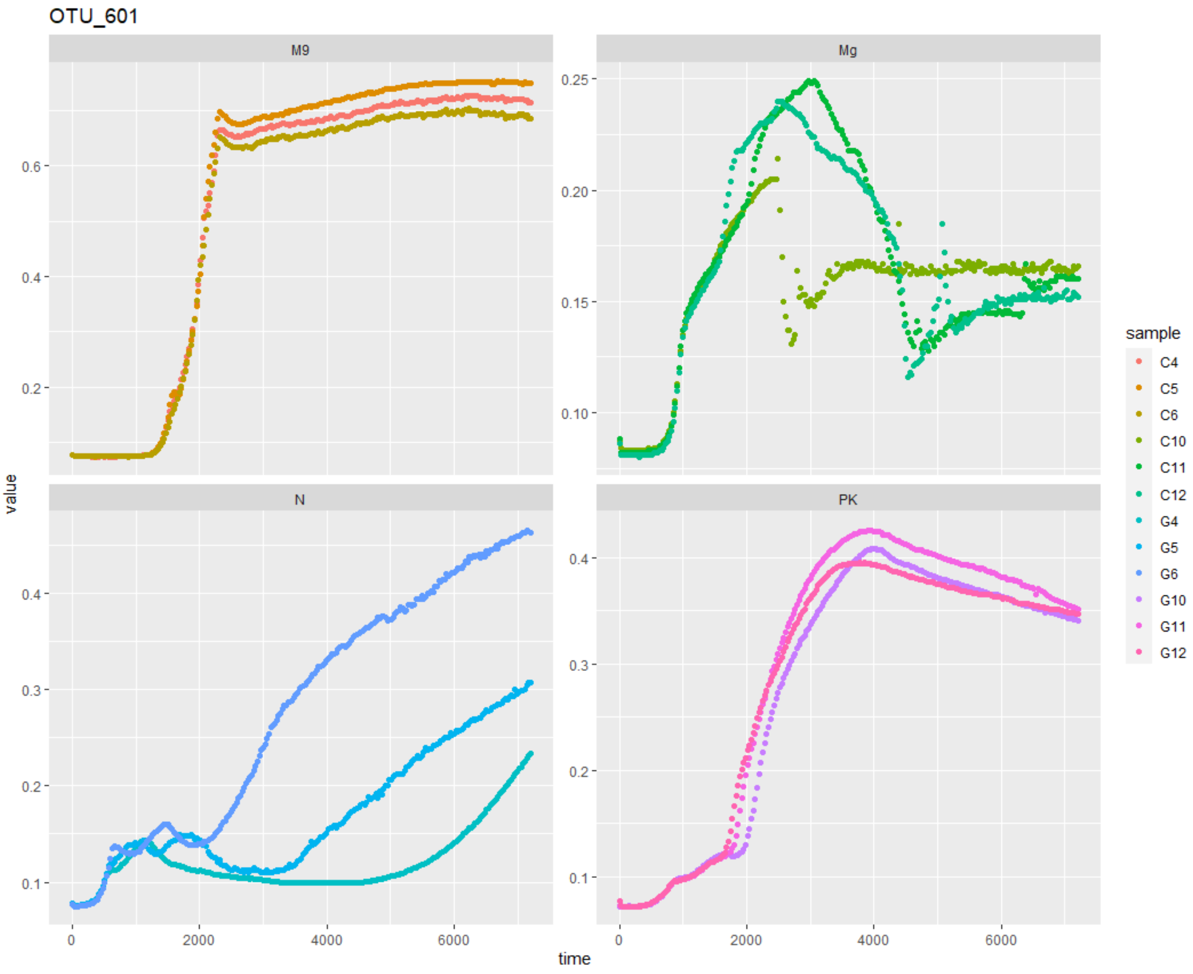

Supplemental Information 4: Growth rate of the selected strain of the genus *Rhodococcus* under nutrient scenarios during pre-experiments plotted against time. Growth rate r is lowest under non-N-scenario

Supplemental Information 5: Fasta-file of the 16S rRNA gene sequence of the bacterial strain used in the experiments. File can be found in a separate document.
